# 

**Published:** 1874-02

**Authors:** 


### TABLE OF CONTENTS.

ORIGINAL COMMUNICATIONS.

Astigmatism. By Swan M. Burnett, M.D. : : : : : : 63
Chloroform and Ether. By J. H. Weir, M.D. : : : : : : 76
Gelseminum. By G. D. Hodge, M.D. : : : : : : : 80
Pueperal Mania. By Thomas H. Mayo, M.D. : : : : : : 82
Some Farther Thoughts on the Uses of "Phylotacca Decandra." By C. H. Tidd, M.D : : : : : : : : : : : : 85

EDITORIAL EXTRACTS AND GLEANINGS.

Malvaviscus Drummondii, Spanish Apple; Modiola Multifida; Liquidambar Styraciflua; Bites of Snakes and Stings of Scorpions, Centipedes, Insects, Spiders, etc.; Small-pox in Houston, Texas; Prophylactic Treatment of Intermittent Fever; Cerebro-spinal Miningitis; Medical Signs and Doses; A New Method of Producing Local Anæsthesia; Vehicle for the Administration of Chloroform. By A. R. Kilpatrick, M.D. : : 88
Cerebal Paresis Treated with Phosphorous; Treatment of Cancrum Oris; The Treatment of Pruigo and Prutis by Carbolic Acid; Phosphorous in Neuralgia. By C. H. Tidd, M.D. : : : : : : : : 92
Needle Swallowed by a Child—Removed Six Months Afterwards from the Thigh; Resection at Hip-joint; Fracture of Femur; Croton-Chloral Hydrate; The Elastic Ligoture; Syphilitic Urethral Discharges; Foreign Bodies in the Air Passages; Ferruginous and Nerve Tonic. By T. Curtis Smith, M.D. : : : : : : : : 93
Treatment of Sore Nipples; Exudations of Diphtheria and Croup; An Anti-Miasmatic Tree; Another Test for Morphia; New Test for Albumen; Glanular Lids; Adulterations of Tea; How to Give Phosphorus; Liquid Nourishment for Sick Stomach; Spermatorrhœa. By Swann M. Burnett, M.D. : : : : : : : : 96
Hemorrhage in Abortion; Impassable Stricture; The Bavarian Dressing for Fractures; Eczema Genitale; Fatal Suffocation from Nitrous Oxide Gas; Veratrum in Inflammation; Sarracenia Purpura; Taraxacin. By R. C. Word, M.D. : : : : : : : : 100

EXTRACTS AND GLEANINGS BY THE LOCAL EDITORS.

Pain an Index to treatment; Purulent Conjunctivitis; The Permanence of Syphilis; On Continuous Discharges After Delivery; New Method of Treating Functio nal Dispepsia, Anœma and Chlorosis; Treatment of Glandular Affections; Sykosis; Saline Cathartics; Hooping Cough; Spermatorrhea; Pelvic Celluitis; Powdered Coal-Tar for Wounds; Chronic Spleintis; Indications for Podophillin; Chronic Chills; Nature of Mumps; Eucalyptus; Topical Use of Chloral Hydrate in Bed Sores, 105

EDITORIAL AND MISCELLANEOUS.

Notices; Premiums, : : : : : - : : 116
Scientific American; Eclectic Magazine; The Poet's Gift of Consolation to Sorrowing Mothers, : : : : : : : 118
New Books; The Galaxy for March is a Splendid Number, : 119
The International Review; Dr. B. T. G.; Dr. C. E. J.; Medical Lexicon, : 120
The Puerperal Diseases, : : : : : : : 121
St. Nicholas; Wilson's Herald of Health and Farm and Household Help; List of Cash Payments to the Record, : : : : 124

